# Survival Nomogram for Curatively Resected Korean Gastric Cancer Patients: Multicenter Retrospective Analysis with External Validation

**DOI:** 10.1371/journal.pone.0119671

**Published:** 2015-02-27

**Authors:** Bang Wool Eom, Keun Won Ryu, Byung-Ho Nam, Yunjin Park, Hyuk-Joon Lee, Min Chan Kim, Gyu Seok Cho, Chan Young Kim, Seung Wan Ryu, Dong Woo Shin, Woo Jin Hyung, Jun Ho Lee

**Affiliations:** 1 Gastric Cancer Branch, National Cancer Center, Goyang, Gyeonggi-do, Republic of Korea; 2 Biometric Research Branch, Division of Cancer Epidemiology and Prevention, National Cancer Center, Goyang, Gyeonggi-do, Republic of Korea; 3 Department of Surgery, Seoul National University College of Medicine, Seoul, Korea; 4 Department of Surgery, Dong-A University College of Medicine, Busan, Korea; 5 Department of Surgery, Soonchunhyang University Bucheon Hospital, Bucheon, Gyeonggi-do, Republic of Korea; 6 Department of Surgery, Chonbuk National University Medical School, Jeonju, Jeollabuk-do, Republic of Korea; 7 Department of Surgery, Keimyung University School of Medicine, Daegu, Republic of Korea; 8 Department of Surgery, Bundang Jesaeng Hospital, Seongnam, Gyeonggi-do, Republic of Korea; 9 Department of Surgery, Yonsei University College of Medicine, Seoul, Republic of Korea; Duke Cancer Institute, UNITED STATES

## Abstract

**Background:**

A small number of nomograms have been previously developed to predict the individual survival of patients who undergo curative resection for gastric cancer. However, all were derived from single high-volume centers. The aim of this study was to develop and validate a nomogram for gastric cancer patients using a multicenter database.

**Methods:**

We reviewed the clinicopathological and survival data of 2012 patients who underwent curative resection for gastric cancer between 2001 and 2006 at eight centers. Among these centers, six institutions were randomly assigned to the development set, and the other two centers were assigned to the validation set. Multivariate analysis using the Cox proportional hazard regression model was performed, and discrimination and calibration were evaluated by external validation.

**Results:**

Multivariate analyses revealed that age, tumor size, lymphovascular invasion, depth of invasion, and metastatic lymph nodes were significant prognostic factors for overall survival. In the external validation, the concordance index was 0.831 (95% confidence interval, 0.784–0.878), and Hosmer-Lemeshow chi-square statistic was 3.92 (*P* = 0.917).

**Conclusions:**

We developed and validated a nomogram to predict 5-year overall survival after curative resection for gastric cancer based on a multicenter database. This nomogram can be broadly applied even in general hospitals and is useful for counseling patients, and scheduling follow-up.

## Introduction

Gastric cancer is the fourth most common cancer, and approximately one million new cases are diagnosed annually worldwide [1]. Although the incidence has decreased substantially, gastric cancer remains the second leading cause of cancer-related deaths in the world and the most common cancer among Korean males [2,3].

The prognosis of gastric cancer patients is mainly associated with the extent of disease. The American Joint Committee on Cancer (AJCC) has developed a staging system to classify gastric cancer into eight risk groups based on the depth of invasion, the number of metastatic lymph nodes, and distant metastasis [4]. Generally, this staging system is strongly correlated with survival; however, different prognoses were also observed among patients at the same pathological stage. These differences may be due to other prognostic factors such as age, sex, tumor size, histological type, and adjuvant chemotherapy, all of which could affect overall survival. Therefore, a more refined method for predicting individualized survival of gastric cancer patients is required, and a nomogram is a good method for this purpose.

A nomogram was initially established by Kattan et al.[5] in 2003. They analyzed 1039 patients’ clinicopathological data and developed a nomogram predicting 5-year disease-specific survival after R0 gastric cancer resection at a single US institution. This nomogram showed superior discrimination to the American Joint Committee on Cancer (AJCC) stage grouping, and was validated using two European cohorts [6,7]. However, Strong et al.[8] observed different survivals between the Unites States and Korea. Even though several different clinical variables were adjusted in the multivariate model, same result was revealed. This result indicated that a different nomogram was required for Eastern gastric cancer patients.

Recently, two nomograms were developed based on the Korean database [9,10]. These nomograms are valuable because they were derived from data collected in Eastern countries, and one was validated by an independent data set (Japanese database). However, it is questionable whether these nomograms can be applied to general hospitals because data originating from a single high-volume center may be biased in terms of treatment outcomes and survival.

The aim of the current study was to develop and validate a nomogram to predict the overall survival of patients who underwent curative resection for gastric cancer based on multi-center data. Korean institutes of various scales participated in the current study, and an independent external validation was performed.

## Materials and Methods

### Study cohort and data

Between January 2001 and December 2006, a total of 3,284 patients underwent conventional open gastrectomy for gastric cancer by nine surgeons from eight institutions (Chonbuk National University Hospital, Dong-A University Hospital, Jaeseng Hospital, Keimyung University Hospital, Seoul National University Hospital, Soonchunhyang University Hospital, Yonsei University Severance Hospital, and the National Cancer Center). Among these patients, we excluded 352 patients who underwent R1 or R2 resection, 392 patients with missing clinicopathological characteristic data, 432 patients with no information regarding adjuvant chemotherapy, and 96 patients with no survival data. Lastly, 2012 patients were included in this study.

The database reviewed retrospectively consisted of patients’ age, sex, pathological characteristics (size, location, histological type, lymphovascular invasion, number of harvested lymph nodes, depth of invasion, lymph node metastasis), treatment-associated factors (extent of lymph node dissection, adjuvant chemotherapy), and follow-up period with survival status. The tumor size was measured at the widest diameter and grouped as less than 5 cm, from 5 cm to 9.9 cm, and more than 10 cm. The tumor location was categorized as upper, middle, lower one-third, and overlapping based on the center of the main lesion. Overlapping is defined that cancer extents over more than two one-third. Regarding histological type, differentiated type included papillary, well-differentiated and moderately-differentiated tubular adenocarcinoma. Undifferentiated type included poorly-differentiated tubular adenocarcinoma, signet ring cell carcinoma, mucinous adenocarcinoma and other special types such as squamous adenocarcinoma, and hepatoid carcinoma. The depth of invasion and lymph node metastasis were categorized according to the 7^th^ AJCC tumor-node-metastasis (TNM) classification.^4^ The extent of lymph node dissection was classified as D1 plus and D2 according to the Japanese treatment guidelines [11].

After surgery, the patients were followed up regularly with physical examinations, laboratory tests (including evaluation of the tumor markers carcinoembryonic antigen and carbohydrate antigen 19–9), chest radiography, endoscopy, and computed tomography. These examinations were performed every 6 months during the first 3 years and annually for the next 2 years [12]. When metastasis was suspected, further evaluations were performed, such as positron emission tomography, bone scan, endoscopic biopsy, and fine-needle aspiration. The follow-up period was calculated from the day of surgery to the last follow-up date, and National Statistical Office data were used for patients who were lost to follow-up.

### Development and validation sets

For development of the nomogram and independent external validation, eight institutes were randomly divided into two groups. Six institutions were assigned to the development set (n = 1,579), and the remaining two institutions were assigned to the validation set (n = 433).

### Statistical analysis

The Cox proportional hazard regression model was used to estimate the hazard ratio (and corresponding 95% confidence interval [CI]) for each of the potential risk factors. Three variable selection methods (forward, backward, and stepwise; inclusion and exclusion criteria of type I error = 0.1 based on likelihood ratio tests) were considered in the multivariate model to build the risk prediction model.

The developed models were validated with respect to their discrimination ability using C-statistics and their calibration ability using Hosmer-Lemeshow (H-L) chi-square statistics. Discrimination refers to the ability of a model to correctly distinguish non-events and events, and it can be quantified by calculating the C-statistic developed for the survival model [13]. The C-statistic is a concordance measure analogous to area under the receiver operating characteristic (ROC) curve.

Calibration measures how closely the predicted probabilities agree numerically with the actual outcomes and a H-L chi-square statistic was used for this purpose [13]. This chi-square statistic was calculated by first dividing the data into 10 groups (deciles) based on the predicted probabilities produced by the model in ascending order. Then, for each decile, the average predicted probabilities were compared to the actual event rate estimated by the Kaplan—Meier approach.

P values were two-sided, and values of < 0.05 were considered statistically significant. All data were analyzed using SAS version 9 (SAS Institute Inc., Cary, NC, USA) and a nomogram was generated based on the multivariate prediction model using R software. All of the results were interpreted by a biostatistics specialist (BH Nam).

### Ethic Statement

This study was performed with the approval of the institutional review boards of the 8 institutions. (National Cancer Center, NCCNCS-13–830; Seoul National University Hospital, H-1407–031–592; Dong-A University Hospital, 14–149; Soonchunhyang University Bucheon Hospital, SCHBC 2014–07–011; Chonbuk National University Hospital, CUH 2012–01–003–001; Keimyung University Dongsan Medical Center, DSMC 2014–07–058; Bundang Jesaeng Hospital, 14–01; Yonsei University Severance Hospital, 2014–1194–001). The participants’ informed consent was waived by each institutional review boards because this study involved routinely collected medical data that were anonymously managed in all stages, including stages of data cleaning and statistical analyses.

## Results

### Clinicopathological characteristics of the development and validation sets

The clinicopathological characteristics of the development and validation sets are shown in Table 1 and S1 Dataset. A majority of the patients underwent D2 lymph node dissection (92.2% and 91.7% in the development and validation sets, respectively) and more than 15 lymph nodes were dissected in most cases (97.5% and 97% in the development and validation sets, respectively). The proportion of patients receiving adjuvant chemotherapy was considerably different between the two sets (38.8% vs. 83.1% in the development and validation sets, respectively). It was revealed that even early gastric cancer patients received oral chemotherapy agents after the operation in one hospital belonging to the validation set.

**Table 1:** Demographic and Clinicopathological Characteristics of the Development and Validation set.

Factors	Subgroup	Development set(n = 1,579)	Validation set(n = 433)
		No of patients (%)	No of patients (%)
Age (year)	< 40	115 (7.3)	26 (6.0)
	40–49	257 (16.3)	76 (17.6)
	50–59	382 (24.2)	106 (24.5)
	60–69	547 (34.6)	151 (34.9)
	≥70	278 (17.6)	74 (17.1)
Sex	Male	1,079 (68.3)	311 (71.8)
	Female	500 (31.7)	122 (28.2)
Tumor size (cm)	< 5.0	854 (54.1)	318 (73.4)
	5.0–9.9	582 (36.9)	100 (23.1)
	≥10.0	143 (9.1)	15 (3.5)
Location	Upper	256 (16.2)	43 (9.9)
	Middle	512 (32.4)	95 (21.9)
	Lower	771 (48.8)	293 (67.7)
	Whole	40 (2.5)	2 (0.5)
Histological type	Differentiated	625 (39.6)	208 (48.0)
	Undifferentiated	954 (60.4)	225 (52.0)
Lymphovascular invasion	Absent	812 (51.4)	301 (69.5)
	Present	769 (48.6)	132 (30.5)
pT	1	593 (37.6)	243 (56.1)
	2	597 (37.8)	119 (27.5)
	3	346 (21.9)	65 (15.0)
	4	43 (2.7)	6 (1.4)
pN	0	797 (50.5)	287 (66.3)
	1	129 (8.2)	31 (7.2)
	2	84 (5.3)	11 (2.5)
	3	569 (36.0)	104 (24.0)
Extent of lymph node dissection	D1 plus	123 (7.8)	36 (8.3)
	D2	1,456 (92.2)	397 (91.7)
No. of harvested lymph node	< 15	39 (2.5)	13 (3.0)
	15–29	406 (25.7)	117 (27.0)
	30–44	615 (39.0)	162 (37.4)
	≥45	519 (32.9)	141 (32.6)
Adjuvant chemotherapy	No	966 (61.2)	73 (16.9)
	Yes	613 (38.8)	360 (83.1)

### Risk factors for overall survival and development of the nomogram

The mean follow-up period for the development set was 51.7 ± 23.5 months (median, 52.0 months), and 351 (22.2%) patients died during the follow-up period.

In the univariate analysis, age, tumor size, location, histological type, lymphovascular invasion, depth of invasion, lymph node metastasis, extent of lymph node dissection, and adjuvant chemotherapy were significantly associated with overall survival (Table 2). In contrast, sex and number of harvested lymph nodes did not have significant effects. Multivariate analyses were performed using the significant risk factors determined in the univariate analysis, and old age, large tumor size, the presence of lymphovascular invasion, advanced depth of invasion, and many metastatic lymph nodes were revealed as significant independent factors for overall survival.

**Table 2:** Risk factors for overall survival according to Cox proportional hazards regression model.

Factors	Subgroup	Univariate analysis			Multivariate anlaysis*		
		Hazard ratio	95% CI	*P*	Hazard ratio	95% CI	*P*
Age (year)	< 40	1			1		
	40–49	1.09	0.67–1.78	0.720	1.09	0.67–1.78	0.729
	50–59	0.70	0.43–1.14	0.153	0.68	0.42–1.11	0.122
	60–69	1.19	0.77–1.86	0.436	1.24	0.79–1.94	0.357
	≥70	1.88	1.19–2.97	0.007	1.63	1.02–2.59	0.040
Sex	Male	1					
	Female	0.96	0.76–1.20	0.712			
Tumor size (cm)	< 5.0	1			1		
	5.0–9.9	2.80	2.20–3.56	<0.001	1.16	0.89–1.53	0.275
	≥10.0	5.43	4.01–7.36	<0.001	1.58	1.11–2.24	0.011
Location	Upper	0.89	0.65–1.22	0.470	0.74	0.53–1.01	0.059
	Middle	0.91	0.71–1.16	0.439	0.81	0.63–1.04	0.101
	Lower	1			1		
	Whole	4.12	2.70–6.27	<0.001	1.57	0.97–2.53	0.067
Histological type	Differentiated						
	Undifferentiated	1.59	1.27–2.00	<0.001			
Lymphovascular invasion	Absent	1			1		
	Present	4.89	3.78–6.34	<0.001	1.58	1.17–2.14	0.003
pT	1	1			1		
	2	4.15	2.85–6.03	<0.001	1.79	1.16–2.76	0.009
	3	11.24	7.80–16.20	<0.001	3.11	1.97–4.92	<0.001
	4	13.89	8.26–23.37	<0.001	3.73	2.11–6.96	<0.001
pN	0	1			1		
	1	2.41	1.43–4.07	0.001	1.45	0.84–2.50	0.183
	2	4.06	2.47–6.67	<0.001	1.98	1.16–3.38	0.012
	3	8.87	6.61–11.90	<0.001	3.40	2.34–4.94	<0.001
Extent of LN dissection	D2	1			1		
	D1 plus	1.44	1.02–2.04	0.038	1.40	0.97–2.02	0.071
No. of harvested LNs	<15	1					
	15–29	1.41	0.57–3.49	0.456			
	30–44	1.92	0.79–4.69	0.151			
	≥45	2.13	0.87–5.21	0.098			
Chemotherapy	No	1					
	Yes	3.42	2.74–4.26	<0.001			

* Backward variable selections methods was conducted with selection criteria of 0.2. Sex, extent of lymph node dissection, No. of harvested lymph nodes were excluded in the multivariate analysis because of no significant effect in the univariate analysis.

CI, confidence interval; LN, lymph node.

**Fig 1:**
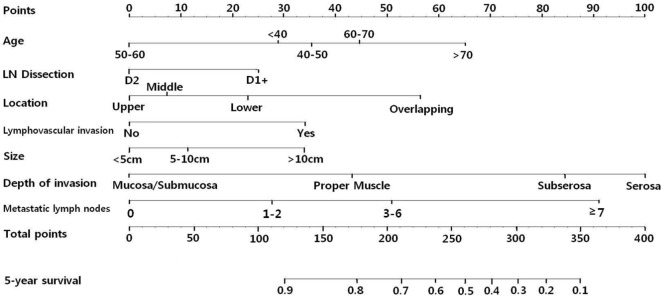
Nomogram to predict 5-year overall survival after curative surgery for gastric cancer. Each clinicopathological factor corresponds to a specific point by drawing a line straight upward to the Points axis. After sum of the points is located on the Total Points axis, the sum represents the probability of 5-year survival by drawing straight down to the 5-year survival axis.

Based on these results, we developed a prediction model and a nomogram predicting 5-year overall survival was generated (Fig. 1). Location and extent of lymph node dissection had no significance in the multivariate analysis, however, they were included in the nomogram according to the exclusion criteria of 0.1. Each clinicopathological factors corresponds to a specific point by drawing a line straight upward to the Points axis. After sum of the points is located on the Total Points axis, the sum represents the probability of 5-year survival by drawing straight down to the 5-year survival axis. For example, a 45-year-old male (35 points) underwent D2 gastrectomy (0 point) for 7cm sized (12 points) gastric cancer located in the lower one-third of stomach (23 points). In the pathological report, the tumor invaded into subserosa (84 points) with lymphovascular invasion (34 points), and there were five metastatic lymph nodes (50 points). For this example, the total point equals 238, and the suspected 5-year survival is approximately 60%. This calculated value could be used in decision making for treatment plans and patient counseling.

### External validation set and performance

In the validation set, the mean follow-up period was 47.0 ± 16.3 months (median, 49 months), and 55 (12.7%) patients died during the follow-up period.

**Fig 2:**
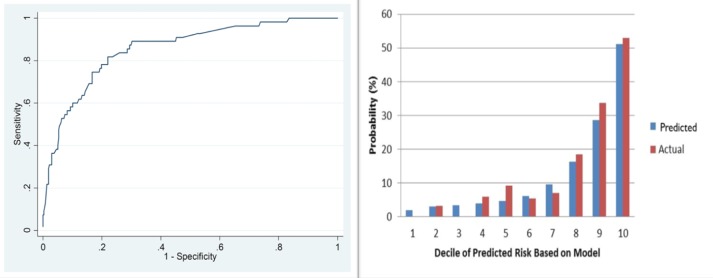
The ROC curves (a) represents the discrimination ability of the model measured by the C-index was 0.831 (95% CI, 0.784–0.878). Calibration plots (b) show the relationship between the predicted probabilities base on the nomogram and actual values of the validation set. The x-axis represents deciles of predicted risk, and the y-axis reveals predicted and actual probability of 5-year survival. The H-L chi-square which measure the calibration was 3.92 (*P* = 0.917).

The external validation was performed by evaluating the performance of the model with respect to its discrimination and calibration abilities. The C-index, which indicated discrimination ability, was 0.831 (95% CI, 0.784–0.878), and the receiver operating characteristic curves are shown in Fig. 2a. The H-L chi-square statistic, which revealed calibration ability was 3.92, and the calibration plot is presented in Fig. 2b (*P* = 0.917).

## Discussion

**Fig 3:**
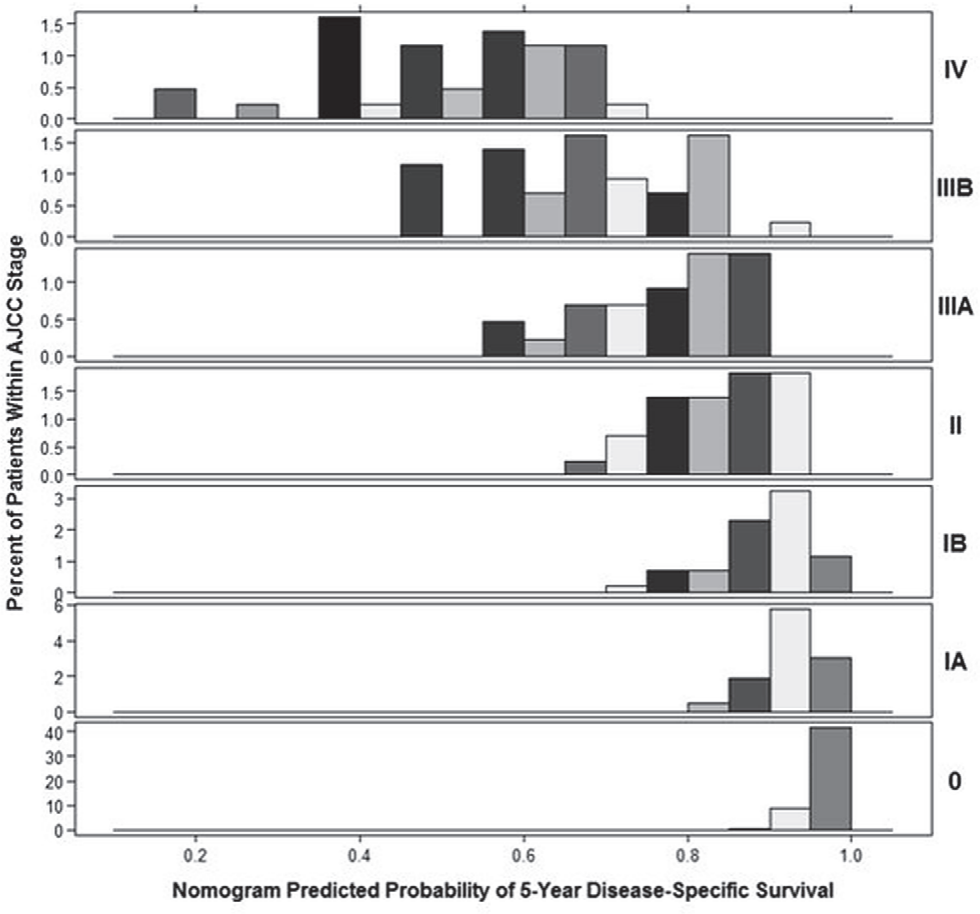
Distribution of nomogram predictions within each AJCC stage grouping.

The TNM staging system is the most common method for predicting patient prognosis. However, considerable survival variation has been observed even in patients at the same stage of gastric cancer. In the current study, we developed a nomogram predicting overall survival after gastric cancer surgery based on a multicenter database, and good performance was shown in the external validation. The advantage of this nomogram over AJCC stage grouping is shown in Fig. 3, the heterogeneity of overall survival within each stage was seen, particularly in the stage IIIA, IIIB, and IV.

As mentioned above, there have been three nomograms predicting survival after gastrectomy for gastric cancer. One is initial US nomogram reported by Kattan et al.[5] in 2003, others were developed by Han et al.[9] and Song et al.[10] based on the Korean database. The main difference between the previous nomograms and the current nomogram is study cohort. Previous nomograms derived from a single high-volume center data. Memorial Sloan-Kettering Cancer Center, Seoul National University Hospital, and the Seoul St. Mary’s Hospital had big database consisted of more than 1000 patients in each hospital. External validation was also performed using other large-volume center database. According to many previous studies, surgical outcomes and survival after gastrectomies were significantly associated with hospital volume [14–17]. Therefore, it is possible that the previous nomograms are limited to tertiary high-volume hospital.

Another difference is that previous Korean nomograms included only D2 gastrectomies. It is well known that D2 lymphadenectomy has been accepted as a standard procedure for patients with resectable gastric cancer [18,19]. However, limited lymphadenectomy is also performed in high risk patient or in complicated situation. Moreover, the recent Japanese treatment guideline recommended D1 plus for early gastric cancer, and in Korea, most early gastric cancer patients undergo D1 plus gastrectomy [11,20–22]. Previous Korean nomograms could not be adapted to patients who underwent limited lymphadenectomy.

On the other hand, the current nomogram derived from various size of hospital database, and the nine involved surgeons collected all their patients’ data from the initial case. Therefore, the current nomogram could be broadly adopted by both tertiary hospitals and general hospitals, and both experienced surgeons and inexperienced surgeons. Moreover, we included both D2 gastrectomy and limited lymphadenectomy, and the proportion of limited lymphadenectomy amounted to 7–8%. Therefore, even patient who underwent limited lymphadenectomy could use the current nomogram for predicting survival.

Some differences are also observed in prognostic factors between previous nomograms and the current one. Kattan et al.[5] revealed that the Lauren classification and the number of negative nodes were significant prognostic factors. Han et al.[9] demonstrated that sex and the number of examined lymph nodes had significant prognostic effects on overall survival. Similarly, sex, gross type, and Lauren classification were independent factors for overall survival in a study by Song et al.[10] However, in the current study, and the number of harvested lymph nodes had no statistical significance even in the univariate analysis. Histological type had a significant effect in the univariate analysis; however, it was eliminated in the multivariate analysis with the selection method. Instead, tumor size and lymphovascular invasion were independent prognostic factors for overall survival in the current study. Considering that the current study was based on multicenter data, we assumed that the number of harvested lymph nodes would vary depending on the surgeons who classified lymph node stations, pathologists, and institutional systems; therefore, the prognostic effect might be decreased.

Tumor location is also one of the different prognostic factors. Kattan et al.[5] and Han et al.[9] demonstrated that upper-third tumors had poor prognosis; however, the current study showed less risk with upper- and middle-third tumors than with lower-third tumors. We reviewed the pathological stage and tumor size according to tumor location, however, could not find any biased relationships causing better prognosis of upper-third tumors. Further study seems to be required.

Recently, large-scale randomized controlled studies demonstrated the survival benefit of adjuvant chemotherapy after curative resection for gastric cancer [23–25]. However, in the current study, adjuvant chemotherapy failed to show statistical significance in multivariate analysis. Various regimens and indications for adjuvant chemotherapy of each institution could influence this negative result. Moreover, interrupted chemotherapy due to adverse effects or patients’ low compliance may reduce the effect of adjuvant chemotherapy on overall survival.

Actually, we compared our nomogram and a nomogrm derived from Seoul National university hospital (SNU nomogram)[9]. The validation set of our data was applied to our prediction model and SNU prediction model, respectively. As a result, C-index value of SNU prediction model was 0.831 (95% CI, 0.783~0.879), which was almost similar to our C-index. However, calibration abilities of SNU prediction model showed that there were significant differences between the predictive and actual survivals (H-L chi statistic = 27.339, p = 0.001). This result supports that our nomogram predicts survival more accurately than SNU nomogram.

Although we have produced good results, the current study had several limitations. Each institution has managed its own database in a different way, and some clinicopathological characteristics were not documented. As a result, 920 (28.0%) of the 3284 patients were excluded due to missing data, which can lead to selection bias. Moreover, the current study only included patients who underwent open gastrectomy. Considering that most patients undergo laparoscopic surgery for early gastric cancer in Korea, we need further evaluation for the application of the current nomogram to laparoscopy-assisted gastrectomy cases.

In summary, we developed a nomogram that predicts individual 5-year survival in patients who underwent curative resection for gastric cancer. This nomogram improves accuracy of survival prediction and can be useful to counsel patients after gastrectomies. Further treatment such as adjuvant chemotherapy can also be decided based on the result of this nomogram. This nomogram was derived from a multicenter database and was validated by an independent external data set. Therefore, this nomogram can be adopted in both tertiary hospitals and local general hospitals.

Finally, validation using a Western cohort will also be needed prior to universal use of the current nomogram. As noted by Strong et al.[8], significant differences in clinicopathological and genetic characteristics between the Eastern and Western cohorts should be considered.

## Supporting Information

S1 Dataset Patients’ clinicopathological dataset. (XLS)
